# Safety of Probiotics: Functional Fruit Beverages and Nutraceuticals

**DOI:** 10.3390/foods9070947

**Published:** 2020-07-17

**Authors:** Irena Žuntar, Zvonimir Petric, Danijela Bursać Kovačević, Predrag Putnik

**Affiliations:** 1 Faculty of Pharmacy and Biochemistry, University of Zagreb, Ante Kovačića 1, 10000 Zagreb, Croatia; izuntar@pharma.hr; 2 Unit of Pharmacokinetics and Drug Metabolism, Department of Pharmacology at the Institute of Neuroscience and Physiology Sahlgrenska Academy at the University of Gothenburg, 40530 Göteborg, Sweden; petric.zvonimir@gmail.com; 3 Faculty of Food Technology and Biotechnology, University of Zagreb, Pierottijeva 6, 10000 Zagreb, Croatia; dbursac@pbf.hr

**Keywords:** probiotic safety, toxicity, pathogenicity, functional food industry, pharmacological interactions, functional fruit juices

## Abstract

Over the last decade, fruit juice consumption has increased. Their rise in popularity can be attributed to the belief that they are a quick way to consuming a dietary portion of fruit. Probiotics added to fruit juices produce various bioactive compounds, thus probiotic fruit juices can be considered as a new type of functional foods. Such combinations could improve nutritional properties and provide health benefits of fruit juices, due to delivering positive health attributes from both sources (fruit juices and probiotics). However, this review discusses the other side of the same coin, i.e., the one that challenges general beliefs that probiotics are undoubtedly safe. This topic deserves more acknowledgments from the medical and nutritional literature, as it is highly important for health care professionals and nutritionists who must be aware of potential probiotic issues. Still, clinical trials have not adequately questioned the safety of probiotics, as they are generally considered safe. Therefore, this reviews aims to give an evidence-based perspective of probiotic safety, focusing on probiotic fruit beverages and nutraceuticals, by providing documented clinical case reports and studies. Finally, the paper deals with some additional insights from the pharmacological and toxicological point of views, such as pharmacological repercussions of probiotics on health.

## 1. Introduction

Probiotics are commonly defined as viable microorganisms [1]. This refers to both dietary supplements and drugs, as well as microorganisms found in fermented foods as a part of human nutrition. They are generally added to foods in order to improve its nutritional value as increased demand for new probiotic products is constantly growing. Probiotics are conventionally added to dairy products, but in recent times, the food industry is trying to develop other food matrices that are suitable for this purpose. Therefore, the formulation of probiotic beverages based on the fruit juices might be a compromise solution as they provide an excellent carrier for these probiotic bacteria. The probiotic strains produce various bioactive compounds, such as vitamins, antioxidants, amino acids and peptides, and when added to fruit juices may offer a synergy of health benefits from both sources. Such products can be considered as a new type of functional foods [2,3]. However, there are concerns regarding the safety of added probiotics to the foods. Hence, the purpose of this review is to provide the perspective of probiotic safety with focus to probiotic fruit (functional) beverages and nutraceuticals.

## 2. Safety and Pathogenicity of Probiotics and Their Assessments

The World Health Organization defines probiotics as “live microorganisms that when administered in adequate amounts confer a health benefit on the host” [4], while in the context of a food product, a minimum of 10^6^ colony forming units per mL (CFU/mL) must be reached if the food product will be labeled as probiotic [5].

Many probiotics on the market usually contain various bacterial strains from different species, rather than a single strain [6]. This is due to the belief that multiple strains of probiotic products will have a greater success of delivering health benefits and broader efficacy, and additional faith that their effect will be an additive, or even synergistic. However, there is one limitation to this type of reasoning. Namely, it is thought that in some cases there can be antagonistic effects between different probiotic species. Even though, this may sound logical and convincing, studies that compared single vs. multispecies probiotics, concluded that such claims are still not clear and should be further investigated [6].

As many bacteria can have an impact on microbial balance in the intestines, only those bacteria species and strains with confirmed positive effects on the host can be selected as probiotics. Hence, on the global market, probiotics that are mostly found include species of *Lactobacillus*, *Bifidobacterium*, *Lactococcus*, and *Enterococcus*. Some other bacteria, such as *Lactobacillus bulgaricus*, or *Streptococcus thermophilus*, are not normally part of intestinal flora, but still, they are categorized as probiotics because of their use as starters in dairy products. The influence of such bacteria on gut microbial balance is believed to be weak, as they lack the colonization properties [7].

Nowadays, probiotics are found (and regulated) in three categories: (i) Foods (fermented foods), with claimed GRAS (generally recognized as safe) status for *Lactobacillus*, *Bifidobacterium*, and *Lactococcus*; (ii) dietary supplements, which are often sold as over-the-counter (OTC) supplements; and (iii) drugs (pharmaceuticals). Categorization depends on probiotic manufacturers and indications of use, but it is also dependent on demands from different regulatory authorities [8,9]. Safety of foods or pharmaceuticals intended for human consumption, including probiotics, is a paramount factor in order to avoid any health hazards. Some clinical studies confirmed that safety of probiotics is apparent, as due to absence of toxicity in diverse populations including: (i) Healthy adult volunteers [10]; (ii) women during late pregnancy and their children during early infancy [11]; (iii) infants aged 0–2 years [12], and children [13]; (iv) hospitalized children [14]; (v) critically ill children [15]; and (vi) immunocompromised patients. After all, probiotics are *a priori* nonpathogenic, meaning, that they are never supposed to cause, or potentiate any disease in humans, regardless of the source of their intake, i.e., foods or OTC supplements.

### 2.1. Probiotics Safety

On the contrary and quite recently, papers from prestigious scientific medical journals, such as Lancet, Cell, England Journal of Medicine, and Nature, started questioning if probiotics are truly safe, as some large-scale clinical trials reported unexpected results [16,17,18,19]. Some researchers stated that many clinical trials of probiotics have clinical data, which are lacking a proper methodology of collecting and interpreting the results, especially regarding the clinical endpoints, besides the lack of the scientific rigor [19]. This recent paradigm shift of apparent probiotic safety (and efficacy) became even more controversial, as seen from Cochrane reviews of 31 trials, where probiotics are recommended as safe (and effective) when used with antibiotics for the treatment of *Clostridium difficile.* However, a new review from only a year later in 2018 [20] concluded that probiotic use (for some diseases), indeed, lacks sufficient evidence, and more research is required to support probiotic recommendations [21,22]. Additionally, Lerner et al. [23] in recent review article also highlighted the safety concerns of probiotic intake and shared the same suspicion with many other authors [24,25], while stating that the list of probiotics’ adverse effects is still underestimated. Therefore, it can be concluded that the safe and effective use of probiotics, from pharmacological and toxicological aspects, seems to be undervalued. On the other hand, it can be often seen how many health professionals, including both, physicians and pharmacists, warmly recommend taking probiotics [26,27]. This is not surprising, considering how much they are advertised, especially to customers and patients, who often, as end-users, have difficulties distinguishing between high and poor-quality products. Namely, probiotic products that contain the same bacterial strains are found to be marketed under various beneficial claims on their labels [28,29].

Probiotics are more frequently categorized as food supplements, and not pharmaceuticals, which implies avoidance of extremely thorough testing which are obligatory for all pharmaceuticals. Unless the probiotic manufacturer makes any specific claims regarding health, probiotics will be classified as food supplements, meaning that the focus on safety c be underestimated. Additionally, if any health claims are found on the packaging of probiotics, regulatory bodies will inspect primarily validity of such claims, and not the safety of the product [30,31,32]. As the global market of probiotic grows, due to rise of probiotic consumption [33], regulatory questions are becoming increasingly apparent, where a framework which is supposed to be uniformly followed by all manufacturers is still lacking. For example, in the EU, Food Products Directive and Regulation (2000/13/EU, 178/2002/EC) regulates both probiotics and food supplements, while European Food Safety Authority (EFSA) oversees reviewing health claims of probiotics, which are usually stated on the label [34]. The EFSA also issues the Qualified Presumption of Safety (QPS) for various bacterial strains. The word “presumption” is the only criteria linked with the actual true safety of probiotics, meaning that safety valuation is not the primary concern. Furthermore, QPS is focused on a healthy population, i.e., the general population, while those at-risk are not included in the assessment [34]. Interestingly enough, EFSA has excluded all health claims for probiotics with the explanation that amassing a healthy gut flora is not a recognized health benefit. At the same time, the regulation in European Union, states that for safety, traceability, and protection of the intellectual rights, every novel food product should have specified strains. Hence, lawmakers stressed the importance of research in determining the safety and toxicity of probiotics [9].

Post-marketing regulatory vigilance is not performed here either, and the term “health benefit”, (often stated on the label) is not a regulated specificity, nor has a clear medical meaning [31,34]. Altogether, it cannot be known if the labels on probiotics are honest, so in the end, it seems justified to have critical thinking about the real efficacy and safety of probiotics. The situation gets even more complex, since there can be so many microorganisms used as probiotics, and there are differences among types of bacterial species, but also among subtypes of the same bacterial species. As a result, probiotics are expected to have different health benefits, but also undesired effects [35,36,37]. Namely, in the host, the survival of microorganisms is variable, where microorganisms can show different effects. Moreover, probiotic fate in the host can be variable, and affected by the differences in probiotic manufacturing and formulations [38], and the probiotic intake source, e.g., foods or dietary supplements [39]. Hence, one of the most important factors, which contributes to better evaluation of probiotic safety as well as probiotic health risk, is a thorough knowledge of the microbial colonization properties [39]. Therefore, probiotics should never be looked at as a uniform group of viable microorganisms [40], as their properties are strain-dependent, i.e., species-specific, but rather on a case-by-case basis, avoiding the one-size-fits-all approach. 

More precisely, potential probiotic health risk can be viewed in two ways [41]. The first way involves the adverse effects of probiotic per se, while the second way involves safety concerns, due to undefined quality standards and manufacturing procedures. Nevertheless, the only standardization of accurate probiotic safety assessment is a retrospective epidemiologic study, accompanied by thorough pharmacological and toxicological post-marketing vigilance [39,42,43] of the product, in order to evaluate further probiotic safety. 

We must remain mindful that consumers can respond in different and often unpredicted ways to any medication, dietary supplement or food (allergic reactions), and probiotics are not the exception. Individual differences, such as age, gender, and underlying pathophysiology, are factors that most contribute to such individual response [44]. Moreover, interactions between genes and food (diet) are nowadays becoming the subject of investigations, because many metabolic pathways are found to modulate the development of many diseases. Furthermore, probiotics, as viable microorganisms, grow and colonize the gut, and in theory, under particular circumstances that could be the source of undesired events in the host and represent a serious health concern [39,43].

Fundamental toxicological and pharmacological concepts of how toxic and therapeutic effects of substances are in almost every case dose-dependent are applicable in the assessment of any apparently safe substances [45]. For instance, common sodium salt (NaCl) or even drinking water if taken in large amounts can be lethal. Hence, probiotics (if taken in critical amounts) can be deadly as well with observable toxicological consequences. However, pharmacological and toxicological interplay regarding the probiotic dose-response relationships are not actually studied—not even in animal models.

For a better assessment of probiotic products, many researchers agree that scrutiny of pathogenicity, infectivity, virulence, metabolic activity, and intrinsic properties are required [39,45,46]. However, additional technological characteristics of the manufacturing process and marketing regulation are definitely needed, due to potential unwanted outcomes. More specifically [42], there is a risk of systemic and local infections, but also risks of unwanted and hazardous metabolic activities, or gene transfer, and antibiotic resistance. Moreover, there is a risk of modulating the host’s immune response due to inappropriate manufacturing process and health risk with possibly lethal consequences [39,42,45,47]. Finally, there is a concern about pharmacological drug interactions with probiotics, which could have clinical significance that is hardly investigated. Hence, to undoubtedly establish the safety and toxicity of probiotics, human clinical trials of probiotics are indeed justified, despite having a high cost [48].

One could also ask about using animal models for the risk assessment of probiotics. Unfortunately, it is shown that such data gave only limited risk assessment, and the response between species is highly variable. Therefore, extrapolating such results from animals to humans, could be quite unreliable [1]. In addition, human clinical trials, as recommended methodological approach, are not without flaws, as there are health risks for study subjects, especially if they are already having health problems [48].

Regarding the safety concerns of probiotic, let us have a look at a systematic review of 17 studies, which included almost 1530 patients with cancer. This review found five cases of bacteremia, fungemia, and positive blood culture tests associated with probiotics, which confirms that patients, even though immunocompromised, are indeed at risk [49]. Few cases reported bacteremia in patients with HIV/AIDS and Hodgkin’s disease after probiotic intake, but such incidences were due to excessive consumption of probiotic-enriched yogurt with *Lactobacillus acidophilus* [50]. Similar observations were also found in animal models [51]. For example, *Lactobacillus gasseri* ATC33323 (purified cell wall fragment), in rats, activated systemic inflammation, and in a higher doses, caused death. Such findings confirmed that the topic of probiotic safety deserves attention, especially if probiotics are intended to be used in critically ill patients, who may have a tendency for unwanted immune modulation and consequently, an inflammatory reaction [52].

### 2.2. Pathogenicity Studies of Probiotics and Clinical Cases

From the 1990s until today, there are reports and clinical cases that described the invasive fungal infections related to *Saccharomyces cerevisiae* (and *Saccharomyces boulardii*) [53,54,55,56]. Even though meta-analysis of probiotics for the prevention of antibiotic-associated diarrhea [56] concluded that *Saccharomyces cerevisiae var. boulardii* is the only effective choice in its management, special caution is still advised in patients with compromised immune function and in those who are critically ill [56].

Besides systemic infections, there are reported cases of local infections as well [42]. The literature describes liver abscess and lung infections. Namely, pneumonia induced by *Lactobacillus*, is noticed even in clinical settings, i.e., under close monitoring of health care professionals. Risk factors that are thought to be responsible for the development of probiotic-induced infections included older age, hepatobiliary disease and diabetes mellitus, patients with a history of malignant diseases, and transplantations [49,57,58].

Probiotics containing *Lactobacillus* are related to the cases of bacteremia [59] and endocarditis [60,61,62] in immunocompromised patients, and in those who have heart defects (with or without prosthetic material). This should not be clinically neglected, as reported mortality from *Lactobacillus* and related endocarditis is 23% [59], and although infections associated with *Lactobacilli* are extremely rare, well-known history of probiotic supplementation is important to exclude probiotics as a cause of endocarditis. Therefore, the use of *Lactobacillus* species, such as *L. rhamnosus*, *L. casei*, *L. acidophilus*, *L. jensenii*, *L. plantarum*, and *L. paracasei*, in immunocompromised patients should be under close monitoring of health care professionals [42,63].

As mentioned earlier, the literature contains conflicting results regarding the positive and negative impacts of probiotics on human health and disease. For instance, one study found that the probiotic intake could not be linked to any negative context of pancreatitis. However, the “PROPATRIA” trial [64] concluded that there were negative impacts and mortality, due to probiotic intake attributable to the bowel ischemia in patients with pancreatitis. In the group of people, who were taking probiotics vs. controls, there was increased mortality due to bowel ischemia. If discussing probiotic induced pathogenicity, this difference was quite significant, i.e., 16% vs. 6%. The proposed mechanism of bowel ischemia is thought to happen due to the increased need for oxygen, after high load of six probiotic strains in these patients. Blood flow was already low, and local inflammation was present; hence, probiotics worsened the clinical picture and led to increased mortality as compared to the control group. As the toxicity of probiotic strain per se, should not be prioritized over the total dose of probiotic, it is clear why it is extremely important not to neglect the dose-response relationship in probiotic combination, as individual toxic responses could be unpredictable.

Sanders et al. speculated about the extent of probiotic colonization, and if there were possible side effects related to a long term of probiotic intake, especially in a population prone to allergies. The consumption of probiotics was related to a higher risk of rhinitis, serious asthma attacks, and atopic dermatitis, allergies and sensitization [65]. On the other hand, other researchers [66,67] demonstrated quite the opposite, where probiotic intake had a positive impact on atopic dermatitis. Nevertheless, in atopic patients, the effects of immunomodulation with probiotics remained to be controversial. However, for such patients, it should be kept in mind that an inadequate immune response can be triggered in some circumstances by any viable microorganisms, including probiotics, as the microenvironment of the host determines the final scenario [59,68].

Some authors reported that the long-term use of probiotics might negatively influence human health and be the cause of antibiotic resistance and higher virulence potential. Probiotics as *Lactobacillus, Lactococcus, and Bifidobacteria* even though as being considered safe and nonpathogenic, in theory, could transfer their antibiotic resistance genes to the opportunistic pathogens, or commensal microflora, with hazardous clinical consequences [69]. Although there are obvious gaps in the current understanding of probiotic resistance to antibiotics, it is demonstrated that *Lactobacillus* species have common intrinsic resistance to tetracycline, vancomycin, and erythromycin. In addition to the reported resistance to streptomycin, clindamycin, gentamicin, oxacillin, and lincosamide. Also, *Bifidobacteria* species showed resistance to tetracycline, streptomycin, erythromycin, gentamicin, and clindamycin, while *Streptococcus* species showed high resistance to tetracycline, ciprofloxacin, and aztreonam [70]. Hence, in one way, probiotics could be considered as a reservoir of resistance while in the case of any probiotic-induced infection, an effective arsenal of antibiotics should be used [71].

Probiotics could be involved in the production of metabolites with toxic potential, as mentioned earlier. One of the possible safety concerns is the production of d-lactate, a compound responsible for the development of d-lactic acidosis [72]. Recently, d-lactic acidosis is related to probiotic consumption, in patients with short bowel syndrome, as well as in infants. *Lactobacillus and Bifidobacterium* are known for fermenting ingested carbohydrates and governing the formation of d-lactate [72,73]. Furthermore, in reported cases of “brain fog” (cognitive impairment), the link between probiotics, d-lactic acidosis (metabolic acidosis), small intestinal bacterial overgrowth (SIBO), and symptoms, such as abdominal bloating, distention, and gas, are not established for sure. However, one study implicated probiotics, as the symptoms of brain fogginess improved when probiotics were discontinued, and when patients received antibiotics. Authors advised caution regarding the excessive use of probiotics, especially in people without any obvious medical reasons, patients suffering from gastrointestinal dysmotility, including the patients who frequently used proton pump inhibitors (PPIs) or opioids [73]. Moreover, there is a case of d-lactic acid encephalopathy, related to the use of probiotics in a 5-year old child (with a history small intestine resection) with a short bowel syndrome [74]. Namely, *Lactomin^®^* is prescribed (in double amount than regularly) for diarrhea two weeks before the child’s neurologic symptoms started to appear. Lactomin^®^ contains *Lactobacillus acidophilis, Lactobacillus bulgaricus, Streptococcus faecalis, and Streptococcus faecium*. In particular, *L. acidophilis* produces d-lactic acid, and it was suggested to be the main cause of d-lactic acid encephalopathy [74].

Some bacteria are able to interfere with amino acids/proteins that can produce potentially toxic substances, such as ammonia, indol, phenols, and biogenic amines [37,45]. This is especially important if such bacteria originated from fermented foods, as it is problematic to estimate the accumulation of these potentially toxic products in the fermentation environment that is difficult to manipulate [75]. To that end, one study reported significant accumulation of cadaverine (a toxic diamine compound, produced by bacterial decarboxylation of lysine), due to the presence of *Lactococcus lactis,* originating from fermented foods [76]. Moreover, biogenic amines, i.e., cadaverine, histamine, or tyramine, from food-fermenting lactic acid bacteria, are known to cause symptoms of severe allergic reactions [77]. Hence, the toxicological significance of consuming fermented foods in larger amounts should be more emphasized [78]. Therefore, current findings regarding the metabolic activity of probiotics and their capacity to produce toxic metabolites, require further clarifications in terms of a real toxicological significance.

Rarely, the use of *S. boulardii* has been related to constipation and increased thirst. Although there are some reports of serious itching rash, fatigue, and pruritus was noticed with some probiotics. It should be noted that fatigue, pruritus, and diarrhea occurred equally in the placebo group as well, so the real toxicological impacts cannot be determined [79,80].

According to some older source of data, probiotics containing *Lactobacillus* were considered as contraindicated in individuals who have a history of hypersensitivity to lactose and milk products [81]. However, recent data provide opposite conclusions, and there are even *Lactobacillus* strains that provide relief for lactose intolerance [82]. Although true that fermented dairy products generally do not contain lactose in the amounts that would be high enough to trigger intolerance reactions in sensitive individuals, still probiotic bacteria can be added to non-fermented dairy products, but their applicability is then limited by the lactose intolerance, milk protein allergies or with diets that require cholesterol restriction [83]. Here fruit juices/beverages are perceived as an alternative because they are healthy and beneficial for all groups of consumers (including vegans and vegetarians), therefore they might be a good nutritional substitute for common dairy foods containing probiotics.

### 2.3. Drug Interactions of Probiotics

The gut microbiota goes through very vibrant, dynamic changes due to constant variations in nutritional status, disease occurrence, pharmacological modulation, circadian rhythms, and natural environmental influences [47]. Currently, it has been accepted that microbiota is having significant impacts on the bioavailability of many drugs and xenobiotics, their pharmacokinetics (PK), i.e., absorption, distribution, metabolism and elimination (ADME). This is additional to the drug efficacy, response and adverse effects, i.e., drug pharmacodynamics (PD) and toxicology. Therefore, probiotics, as a part of the host’s microbiota could affect the “destiny” of many drugs as well [47]. In other words, probiotics could influence the bioavailability of some drugs (defined as “*unchanged drug fraction of an administered dose that enters systemic circulation*”), as well as drug PK/PD (simply defined as “relationship between drug concentration with drug effect”) and toxicity [84,85,86]. Koziolek et al., suggested that changes of the microbiome due to the intake of probiotics need further investigations, as probiotic-drug interactions could be clinically significant and not just speculations.

Earlier, it was thought that a drug absorbed from the gut, cannot interact with the host’s microbiota, except in the case when the drug is manufactured as sustained-release dosage form, or when it is a subject to a liver-intestine interplay, i.e., enterohepatic recirculation, which consequently prolongs pharmacological effect [47]. However, new findings regarding the composition of the small intestine, and biotransformation potential of bacteria in the gut, showed that interactions between probiotics and drugs are real, despite lacking the enterohepatic recirculation, and sustained-release dosage form [47,87].

Clear examples can be seen in rats fed with probiotics, who had a significant increase of amiodarone (antiarrhythmic agent) bioavailability [88]. A similar is noted in diabetic rats after they received gliclazide (antihyperglycemic agent) [89] and in rabbits after the administration of amlodipine (antiarrhythmic agent) [90]. Authors that studied amiodarone [88] proposed that the increase of its bioavailability by almost 43% is due to a decrease of pH in the intestine, which consequently, facilitated ionization of the amiodarone and impacted amiodarone transit. Alternatively, it was speculated that increased uptake was caused by the OATP2B1 (influx transporter) upregulation [88]. Surprisingly, up until now, there is no human data available regarding this topic. Thus, only hypothetical relevance regarding the increased bioavailability of amiodarone can be discussed. In brief, as amiodarone already has a risk of causing serious and life-threatening side effects, any additional increase of its bioavailability could be extremely toxic. 

Regarding gliclazide [91], when probiotics were given to healthy rats, mucosal efflux drug transporters that control its transport were upregulated. In diabetic rats, the opposite occurred, i.e., mucosal influx drug transporters were upregulated, which could have clinical significance for diabetic patients, as their glucose levels must be maintained at a relatively stable concentration. On the other hand, some other studies reported a decrease in drug bioavailability. For instance, tacrolimus (immunosuppressive agent) required higher doses in patients who had higher amounts of *Faecalibacterium prausnitzii* in their fecal samples [92]. 

There are numerous other examples of how gut microflora influences the pharmacokinetics of many drugs, such as digoxin, irinotecan, indomethacin, insulin, levodopa, ketoprofen, lovastatin, risperidone, and sulfasalazine, etc. This could have clinical significance regarding the pharmacological response, safety, and toxicity, especially if the drugs have a narrow therapeutic index. Meaning, that even the smallest increase of drug bioavailability will significantly change drug range of concentrations, regarding the effectivity vs. toxicity, towards toxicity and adverse effects [47]. Therefore, it is realistic to expect in the future more exciting research regarding the probiotics and drug pharmacokinetics and interactions.

Some bacteria can interfere with bile acids, due to their bile salt hydrolase (BSH) enzyme. Additionally, there is another fact regarding bile acids and their salts in this context of interfering with probiotics. Namely, bile acids can modulate the absorption of some drugs, especially poorly soluble ones [93]. Pavlovic et al. [94], and Moghimipour et al. [95] indicated that the bile salts increased membrane permeability and fluidity, which is positively correlated with the fraction of the drug absorbed, i.e., drug bioavailability and delivery. Moreover, by forming micelles, bile salts can affect transcellular absorption and increase both, solubility and dissolution of drugs [94,96]. Further, it would be interesting to determine the influence of probiotics on drug PK/PD, as well as to reveal the clinical influence of bile acids on drug PK.

Regarding the well-known pharmacological interactions “probiotic-antibiotic”, it is recommended to administer antibiotics for at least two hours before/after probiotic bacteria. Similarly, probiotics containing yeasts, such as *S. boulardii*, interact with antifungals. Hence, antifungal drugs, such as clotrimazole, ketoconazole, griseofulvin, and nystatin, are contraindicated with *S. boulardii* [97].

In the discussion of the general toxicity of probiotics, it is already stated how probiotics should be used with caution in patients who are immunocompromised, or in those who are using chemotherapeutic agents or immunosuppressant drugs (cyclosporine, tacrolimus, azathioprine, etc., as this could induce pathogenic colonization (and infection) in these patients [45]. However, there are conflicting reports in these at-risk populations, and currently, clinical trials are evaluating the safety of a few probiotic strains in cancer patients receiving anticancer therapy. It is speculated that probiotics could lower the occurrence of diarrhea and mucositis—serious adverse reactions of anticancer therapy [98,99].

In the end, the field for exploring the nutritional impacts of probiotics and their effects on the host’s health and disease is very broad, unexplored, and interdisciplinary. Thus, very tempting for further scientific investigations by many researchers, coming from different fields of expertise. Many factors are influencing the way of how we look at probiotics, so a “one-size-fits-all” criterion cannot be applied in revealing the missing pieces about their roles, effects, safety, and toxicity. As gut microbial balance can be very easily shifted, so can be the safety paradigm of probiotics.

### 2.4. Assessment of Probiotic Safety

There are a few ways of assessing the safety of probiotics [68]. Particular focus can be placed on the intrinsic (nonpathogenic) properties of different strains and species, their pharmacokinetic (PK) properties, and strain-host interactions. Intrinsic properties, such as bile salt deconjugation properties, mucin degradation properties, or platelet aggregation properties (which seem to be responsible for cardiac valve colonization and formation of unwanted metabolites in experiments), might be hazardous for human health and can be studied in vitro [38,39,40,41,42,43,44,45,46,47,48,49,50,51,52,53,54,55,56,57,58,59,60,61,62,63,64,65,66,67,68,69,70,71,72,73,74,75,76,77,78,79,80,81,82,83,84,85,86,87,88,89,90,91,92,93,94,95,96,97,98,99,100]. As the probiotic survival differs for varies bacterial species, to define the specific strain, collecting feces can be used for studying probiotics in vivo. Other approaches include intestinal intubation or performing mucosal biopsies [38,39,40,41,42,43,44,45,46,47,48,49,50,51,52,53,54,55,56,57,58,59,60,61,62,63,64,65,66,67,68,69,70,71,72,73,74,75,76,77,78,79,80,81,82,83,84,85,86,87,88,89,90,91,92,93,94,95,96,97,98,99,100].

The Food and Agriculture Organization of the United Nations (FAO) and the World Health Organization (WHO) provided guidelines [4] for the evaluation of probiotic safety used in foods. Namely, it is recommended that probiotic strains are characterized by a series of inspections (strain specificity is linked with probiotic effects) which will determine possible health hazard risks. Series of inspections include testing of antibiotic resistance properties, probiotic metabolic activities, and unwanted product formation, e.g., bile salt deconjugation, or d-lactate production. Furthermore, it should be possible to assess adverse effects for consumers by accurate surveillance and epidemiological studies and inspect toxin(s) production and hemolytic activity of probiotics after their intake. Assays should also test probiotic properties in animal models, which will be immunocompromised. Additionally, evaluation of probiotic safety should include tests of anti-mutagenic, anti-carcinogenic, and nonpathogenic probiotic properties [100,101]. Nevertheless, consulting a healthcare expert(s) is always warranted to avoid any issues, regardless of the reasons for probiotic use, especially in cases of serious illness, or hospitalization, which demands close monitoring of patients.

## 3. Probiotics: Functional Foods

**Figure 1:**
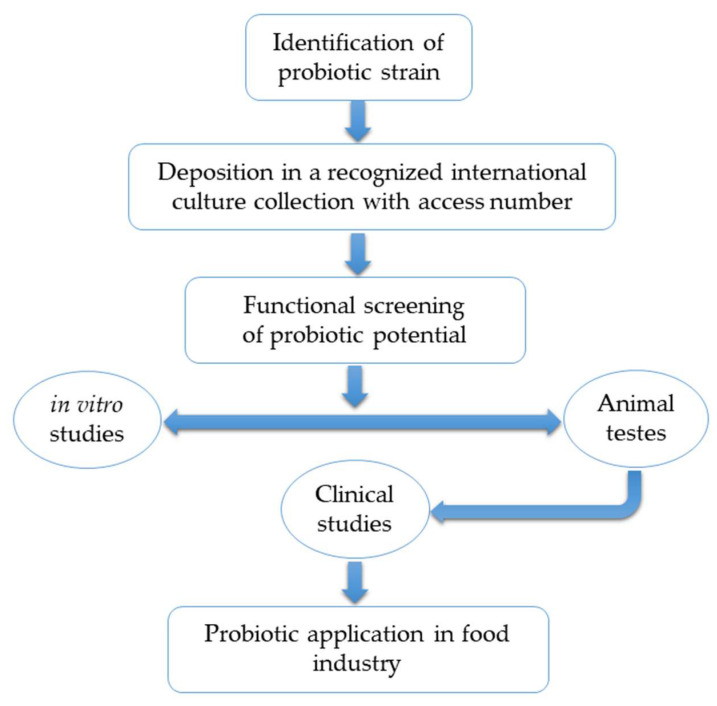
Basic steps for functional probiotic food development.

The value of many foods on the market can be often enhanced by the addition of probiotics. It is not surprising that such foods are considered a better choice in the eyes of the consumers [102] who perceive it as the one with health benefits, so its higher cost is simply justified [103]. Nevertheless, when it comes to probiotics as food components, the whole picture must be looked very carefully as consumers are not homogenous groups, so the assessment of probiotic safety should not be generalized. A detailed review on relevant concerns during functional food development was published elsewhere [104]. However, basic steps in manufacturing and development of probiotic (functional) foods are given in Figure 1.

The most widely used probiotics in the food industry are given in Table 1. Due to the large body of available literature, for further information about the use of various probiotics strains in food industry reader is referred to other sources [79,105,106]. Briefly, strains of *Lactobacillus* and *Bifidobacterium* genera are very heat sensitive [107], therefore *Bacillus coagulans* has attracted the industry interest as this spore-forming bacteria is resistant to heat and possess some characteristics of *Bacillus* and *Lactobacillus* genera [108]. Although probiotics are described as “beneficial” or “friendly” bacteria, however, it should be noted that some types of yeasts, such as *Saccharmyces* (*Saccharomyces cerevisiae* var. *boulardii*; *S. boulardii*), are also defined as probiotics [37,109]. A crucial point of probiotic stability in foods is their ability to remain in high amounts in the product during processing and storage, together with their viability after ingestion [110,111]. Moreover, various sugars, salt, antimicrobials, compounds used as aroma, water content, oxygen level, pH, temperature, and packaging material impact the probiotic viability in both, positive and negative way [81,112].

In comparison to the pure chemicals, substances, or pharmaceuticals, it is harder to predict the impact of probiotic bacteria in foods and their causal relationship regarding the possible adverse effects. Namely, by exploring the hazardous effects of bacteria from food to date, it is obvious that the risks of toxicity are constantly present [38]. For a better understanding, quantitative risk assessment models should be used. However, “minimal infective dose” for monitoring consumer probiotic safety cannot be straightforwardly determined, as there are just too many parameters. For instance, there is a plethora of microbes and various host’s factors, besides the manufacturing process, that influences the probiotic viability. Moreover, there is too large individual variation among consumers, as they can be healthy or with the disease. Additionally, the general cellular mechanism of probiotic effects, safety, and toxicity in humans are still demanding further clarification and studies [38]. In other words, hazards of probiotic intake from food could not be easily toxicologically predictable, especially with inter-individual and intra-individual differences among consumers. Even though theoretical concerns about bacteremia and fungemia are justified, it is unlikely that probiotics from food could show infectivity in a healthy population.

Reported cases of probiotic pathogenicity from foods are related to an immunocompromised people, such as child following bone marrow transplant [113], and 74-year old women with a history of diabetes, who reported a daily intake of 500 mL of dairy drinks containing *L. rhamnosus* GG to relieve her abdominal discomfort [114]. The case of the immunocompromised patient with AIDS, who developed bacteremia from *Lactobacillus acidophilus*, related to excessive consumption of probiotic-enriched yogurts, was already mentioned before [50]. On the positive side, it is interesting to mention that there is some evidence that probiotics can act as potential adsorbents of aflatoxins (ubiquitous contaminants) found in foods [115], and besides, interacting with food components, probiotics recently came in the spotlight, due to the possibility to influence pharmacokinetics/pharmacodynamics of drugs that was previously explained.

### Probiotic Fruit Beverages

The production of fruit juices (contain 100% fruit), nectars (up to 25–99% fruit) and juice drinks (up to 25% fruit content) has become one of the largest sectors in the food industry. According to the European Fruit Juice Association (AIJN), global consumption of both fruit juice and nectars was 36,247 million liters in 2017, while all EU countries together consumed 9187 million liters [116]. In particular, fruit juices contain appreciable amounts of dietary fibers, antioxidants, polyphenols, minerals and vitamins, whereas probiotic addition could further enhance benefits of fruit juices consummation. The particular advantage of fruit juices is that they provide a good environment that is capable of stabilizing probiotic strains [83]. Furthermore, the addition of probiotics improves nutritional properties of fruit juices, and enhances native antioxidant properties of beverages. This is additional to the lowering of the pH in the intestines, which has positive repercussions on digestion, absorption of calcium, iron, and magnesium from the native fruit matrix. This is a very desirable property from nutritional aspects [117,118,119] with supplying ascorbic acid (vitamin C) that has a protective effect on probiotic viability as well [120]. Therefore, such a beneficial combination seems to be an excellent nutritional choice, and it is not surprising that the idea of consumption of such beverages is rapidly growing in the world markets [121,122].

However, there is a question of proper assessment of the bioavailability (fraction of nutrient secreted into circulation and available at the site of action) and bioaccessibility (fraction of bioactive substance that is released from the food matrix) of the health-related bioactive components in the beverages which refers to probiotics as well. Both of these parameters cannot be easily assessed in functional fruit beverages. Secondly, metabolism of health-related bioactive component must also be considered, because sole data of the quantitative input is not sufficient, i.e., the most abundant health-related bioactive compounds from the food matrix do not necessarily imply that it will reach the highest concentrations at the physiological site of the action. For instance, in the case of probiotics, they should be able to survive the exposure to the pepsin [47]. Finally, as seen with medications, absorption of health-related bioactive compounds from food matrix can also differ in the population [48], and it does not help either that there is an evident gap in knowledge about the physicochemical and physiological processes that are involved in the transformation nutrients in the fruit juices [37]. Hence, new techniques and ideas about probiotic functional beverage formulations are more than needed in the near future.

From an industrial perspective, there are always challenges related to adding health-enhancing components, including probiotics, to food matrix. For example, the process of development and formulation of fruit juices as a probiotic carrier is a very complexed task. To design a functional (fruit) beverage with probiotics, it is important that the strains should survive at lower pH [123]. This is additional to resistance to added preservatives and sugars that can negatively influence probiotic viability, and therefore, it is important to examine the stability of probiotic strains in a model juice systems [120]. Usual limitations for the addition of probiotics to fruit juices include: The high acidity, the presence of oxygen, the inadequate amounts of free amino acids, short peptides, and oligosaccharides required for probiotics [124]. Other disadvantages of using probiotics in fruit juices are related to the presence of dyes, flavors, preservatives, antimicrobial components and influence on sensory characteristics [124]. Hence, the proper selection of the right probiotic strain is crucial, as their stability, survival and functionality are more challenging in juices as compared to the addition in common fermented dairy products [125,126,127]. 

On the other hand, there are numerous options for fruit juices that could be suitable as carriers of probiotic bacteria [128,129,130,131]. Suitable fruits as raw materials include: papaya [132], cranberry, lemon, grapefruit, blackcurrant [130], orange [133], apple [134], acerola [127], apple-carrot juice, and pear juice [121]. Some examples of used probiotics in fruit juices are *Bifidobacterium* and *Lactobacillus* species [119], such as *L. plantarum*, *L. acidophilus*, *L. helveticus*, *L. casei*, *L. paracasei*, *L. rhamnosus*, etc. [135]. To alieve manufacturing limitations, some of the proposed approaches [136] to promote probiotic survival in fruit juices are microencapsulation [127,137], fortification with additional prebiotics [138], probiotic strain exposure to the sub-lethal stress which induces adaptive stress response and survival [125], refrigeration and additional use of antioxidants, such as vitamins [139].

It is also important to mention common authenticity issues regarding the use of fruit juices due to various potential frauds including: Water and sugar addition; partial replacement of fruit juice by juices made from concentrates; added products from undeclared cheaper fruits; addition of undeclared ascorbic acid/vitamin C; addition of undeclared organic acids (e.g., citric acid and malic acid); addition of flavor compounds (natural or synthetic); colorings (e.g., anthocyanin extracts, cochenille red, beetroot); adding the texture influencing agents (e.g., pectin). Moreover, the addition or over-proportional use of fruit extracts, which were produced by unauthorized technology and declaration of false origins or declaration of deceitful fruit varieties [140]. In general, it can be stated that most fraud has no real impact regarding the consumers’ safety. However, every food fraud could be a potential health risk, especially in the case of contamination with unexpected agrochemicals [141] or contaminants, even from the probiotic supplementation [142].

Altogether, the recent developments of food processing technologies and constant demands from the consumers regarding the more nutritious and safe food products, fruit juices with added probiotics are soon expected to become a new class of functional foods and important element on general food markets, as well as an integral part of proper nutrition [143,144]. This drive is additionally fostered by an increase in vegetarianism where some companies already have probiotic fruit juice beverages in their portfolio for those seeking a healthy lifestyle [83,84,85,86,87,88,89,90,91,92,93,94,95,96,97,98,99,100,101,102,103,104,105,106,107,108,109,110,111,112,113,114,115,116,117,118,119,120,121,122,123,124,125,126,127,128,129,130,131,132,133,134,135,136,137,138,139,140,141,142,143,144,145].

## 4. Conclusions

An enlarged interest of food industry to find new probiotics non-diary vehicles led to increased use of fruit juices as new matrices, representing a new type of functional foods with a great potential for providing even more health benefits for the consumers and for those seeking a healthy lifestyle. Design of functional fruit beverages with probiotics is still a challenging task, but with current and future technological solutions, it should be possible to derive nutritional and economic benefits for consumers and industry from these types of product.

Probiotics do not work in the same way for every individual, and they should be consumed considering the probiotic strain(s) specificity and sources of intake, levels of exposure, manufacturing properties, along with demands from regulatory authorities, pathological states, and general nutritional status with a known history of using medications. From the pharmacological and toxicological aspects, probiotic safety and toxicity, along with their efficacy and observed health benefits are dependent on various factors, and there is not a “one-size-fits-all” criterion for their clinical evaluation and recommendations of intake. Hence, we are suggesting individualized clinical evaluation before any consumption of probiotics. In general, probiotics are considered as safe for a healthy population, but they may pose a threat for at-risk populations, especially if considering documented case reports and theoretical concerns about their safety and toxicity. Regardless of the age, at-risk populations include critically sick patients, patients at intensive care units, postoperative and hospitalized patients, and especially immunocompromised patients. Besides supporting the idea about long-term clinical studies of probiotics, we expect that questions of probiotic efficacy, safety, and toxicity for humans will be the focus of future research focus, and provide the missing pieces of the puzzle needed for defining probiotics as aid or detriment to the health.

## Figures and Tables

**Table 1:** The most widely used probiotics in the food industry.

Lactobacillus Species	Bifidobacterium Species	Others
*L. acidophilus*	*B. adolescentis* *B. animalis* *B. breve* *B. bifidum* *B. infantis* *B. lactis* *B. longum*	*Bacillus coagulans*
*L. amylovorus*		*Bacillus cereus*
*L. brevis*		*Clostridium botyricum*
*L. casei*		*Enterococcus faecalis*
*L. rhamnosus*		*Enterococcus faecium*
*L. crispatus*		*Escherichia coli*
*L. delbrueckii subsp. Bulgaricus*		*Lactococcus lactis subsp. Cremoris*
*L. fermentum*		*Lactococcus lactis subsp. Lactis*
*L. gasseri*		*Leuconostoc mesenteroides subsp. Dextranicum*
*L. helveticus*		*Pediococcus acidilactici*
*L. johnsonii*		*Propionibacterium freudenreichii*
*L. lactis*		*Saccharomyces boulardii*
*L. paracasei*		*Streptococcus salivarius subsp. Thermophilus*
*L. plantarum*		*Sporolactobacillus inulinus*
*L. reuteri*		
*L. salivarius*		
*L. gallinarum*		
